# Skeletal muscle knockout of NAD(P)H oxidase 2 delays the development of isotonic diaphragm fatigue in mice

**DOI:** 10.1016/j.freeradbiomed.2025.08.045

**Published:** 2025-08-21

**Authors:** Ravi A. Kumar, Vinicius Mariani, Chaoran Yang, Leonardo F. Ferreira

**Affiliations:** a King’s College London British Heart Foundation Centre of Excellence, School of Cardiovascular Medicine & Sciences, London, United Kingdom; b Department of Orthopaedic Surgery, Duke University School of Medicine, Durham, NC, USA; c Department of Physics, Duke University, Durham, NC, USA

**Keywords:** NADPH oxidase, Diaphragm, Fatigue, Power, Isotonic, Contraction

## Abstract

Mechanisms of skeletal muscle fatigue are commonly studied under isometric conditions, which exclude muscle shortening and limit physiological relevance. We developed a novel *in vitro* protocol to examine isotonic fatigue using afterload contractions that permits the study of additional active (velocity, power, work) and passive (stiffness, energy loss) mechanical properties of muscle. During the development of this protocol, we examined the impact of shortening load during afterload contractions on the development of fatigue, and observed a relationship where fatigue onset is more rapid and severe with larger shortening loads (30 % vs. 45 % vs. 60 % maximal isometric force). We then applied this protocol to investigate the contribution of NAD(P)H Oxidase 2 (Nox2) to fatigue development and recovery. Nox2 was deleted from skeletal muscle using the Cre-LoxP system (skmNox2KO), while Cre-negative littermates were used as controls. Knockout of Nox2 attenuated the decline in power and increased total isotonic work performed during repeated contractions compared to controls. Recovery kinetics of power, work, and isometric force were similar between groups. Passive mechanical properties—including stiffness and energy loss— increased with fatigue but were unaffected by Nox2 deletion. These findings highlight the importance of incorporating isotonic contractions to uncover fatigue mechanisms and suggest that Nox2, and presumably reactive oxygen species, contributes to the decline in muscle power during repetitive shortening contractions.

## 1. Introduction

Fatigue describes a decline in the contractile output of skeletal muscle from repetitive use that is recoverable with time [1,2]. Peripheral muscle fatigue, specifically, refers to a decline in contractility due to events occurring at or distal to the neuromuscular junction. Repeated contractions also impact passive viscoelastic properties of skeletal muscle [3–5], which influences joint stability and the susceptibility of athletes to injuries [6–8]. Improved understanding and subsequent mitigation of factors contributing to fatigue can improve injury prevention and performance in competition for athletes, or the quality of life for patients who suffer from accelerated muscle fatigue as a consequence of their disease.

Isolated muscle fibers, bundles or groups are a valuable model to investigate mechanisms of fatigue. Traditional experimental approaches have largely relied on isometric contractions in which the ends of a muscle (or muscle groups) are held at a fixed length and force is recorded following repeated electrical stimulation, with careful consideration to stimulation frequency and duty cycle. Isometric contractions can be implemented easily *in vitro* but this approach neglects muscle shortening, a fundamental aspect of muscle contraction that influences metabolism and fatigue. Muscle shortening against a load is a feature of isotonic contractions, where the output of muscle force and velocity enables the calculation of power (W) and work (J). Development of mechanical power and performance of work are essential for most practical applications of skeletal muscle (e.g. limb movement). Mechanisms of fatigue also appear different with dynamic versus static/isometric contractions [9–12] and the type of contraction may also differentially impact passive mechanical properties. Thus, an element of muscle shortening should be considered when investigating mechanisms of fatigue.

One notable contributor to peripheral muscle fatigue is the accelerated production and accumulation of reactive oxygen species (ROS) following repetitive contractions [1]. ROS signaling and oxidation occurs within microdomains close to the source of production. While there is strong evidence of causality between increased oxidant accumulation and decline in muscle function (e.g. Refs. [13–16]), few studies have examined distinct ROS sources as contributors to peripheral muscle fatigue. NAD(P)H Oxidase 2 (Nox2) produces extracellular superoxide (${\text{O}}_{2}^{.-}$) and is a promising candidate of increased ROS production associated with muscle fatigue based on its localization and activation because muscle cells are rich in extracellular superoxide dismustase and the resulting H_2_O_2_ can enter the cell via aquaporin [17]. Nox2 refers to a complex of subunits that, upon activation via the phosphorylation of the activating subunit p47^phox^, assemble to form a transmembrane complex [18]. Immunohistochemical assessment revealed subunit localization to the sarcolemma and t-tubules [19–21]. Previous findings provide strong evidence that Nox2 is a primary source of ROS during skeletal muscle contractions [20,22]. However, muscles from Nox2 deficient mice show fatigue properties similar to wild type controls using isometric fatiguing contractions [23]. The method to study fatigue is of considerable consideration here, such that the impact of key proteins involved in redox homeostasis on muscle function, especially mechanosensitive processes and enzymes, like Nox2 [24], may be overlooked if muscle shortening is not included.

Here, we describe the development of an *in vitro* isotonic fatigue protocol in isolated murine diaphragm strips with potential translational relevance. We then implement this protocol to test the role of skeletal muscle Nox2 contributing to active and passive mechanical properties of peripheral isotonic fatigue using muscle from animals with skeletal muscle knockout of Nox2 and relevant controls.

## 2. Methods

### 2.1. Experimental animals

All procedures performed in animals followed guidelines established by the National Institutes of Health and were approved by the Institutional Animal Care and Use Committee of the University of Florida (Protocol #20189337), the former affiliation of the authors where these experiments were conducted (RAK, VMM, LFF). Animals were housed at our institution’s facilities under a 12-h:12-h light-dark cycle with access to standard chow and water *ad libitum*. Skeletal muscle specific Nox2 knockout (skmNox2^KO^) animals were generated using an inducible Cre-LoxP as previously described [25]. All experimental mice included in this study were male and carried a floxed Nox2 allele [26]. Experimental groups differed by the presence (Cre positive - skmNox2^KO^) or absence (Cre negative - skmNox2) of inducible Cre recombinase under the human skeletal actin promoter (HSA) [27]. At 12–15 weeks of age, all experimental animals received tamoxifen (5 consecutive days, 80 mg/kg body weight dissolved in ethanol, delivered in sunflower oil) through intraperitoneal injections. Recombination and excision of the floxed Nox2 allele only occurred in Cre positive animals, which was confirmed by PCR as previously described [25]. Mice were sacrificed 4–6 weeks after tamoxifen injections for terminal experiments (n = 3 for preliminary experiments testing different isotonic loads; n = 7 skmNox2, n = 9 skmNox2^KO^ to examine the impact of Nox2 on isotonic fatigue). We anesthetized mice with an oxygen-isoflurane gas mixture (5 % induction, 2–3 % maintenance) and proceeded with a thoracotomy to retrieve the entire diaphragm, which was placed in ice cold Tyrode solution (bicarbonate buffer supplemented with glucose; in mM: 121 NaCl, 5 KCl, 0.5 MgCl_2_, 0.4 NaH_2_PO_4_, 24 NaHCO_3_, 1.8 CaCl_2_, 0.1 EGTA and 5.5 D-glucose).

### 2.2. Diaphragm contractile measurements

#### a Diaphragm bundle preparation

We prepared a diaphragm bundle for assessment of *in vitro* contractile function as described previously [28]. Briefly, one muscle bundle per animal was surgically isolated with portions of the rib and central tendon attached, then mounted to a glass rode and dual mode Muscle Lever System (300C-LR; Aurora Scientific, Aurora, ON, Canada) using 4–0 silk suture. The bundle was placed between two platinum electrodes in a water-jacketed organ bath containing Tyrode solution and continuously gassed with 95:5 % O_2_:CO_2_ at room temperature. Bundle length was adjusted to achieve maximal tetanic tension (optimal length, L_o_) via repeated 120 Hz stimulations (current: 600 mA; pulse width: 0.25 ms; train duration 300 ms) interspersed by 1 min intervals. The bundle was placed at L_o_ and length maintained constant throughout the protocol during the ‘inactive’ phase after stretch-release cycles and preceding each electrical stimulation. Using this approach, the initial tension on each muscle ranged from 4 to 5 mN. Precise length changes (stretch-release cycles) were made with a dual mode Muscle Lever System. The temperature of the organ bath was brought to 37 °C for downstream measurements. We then measured maximal isometric force (F_max_) via a 300 Hz stimulated contraction (current: 600 mA; pulse width: 0.25 ms; train duration: 300 ms). This measurement served as the maximal reference force for subsequent isotonic contractions.

#### b Construction and characterization of the force-velocity curve in afterload contractions

**Fig. 1.**
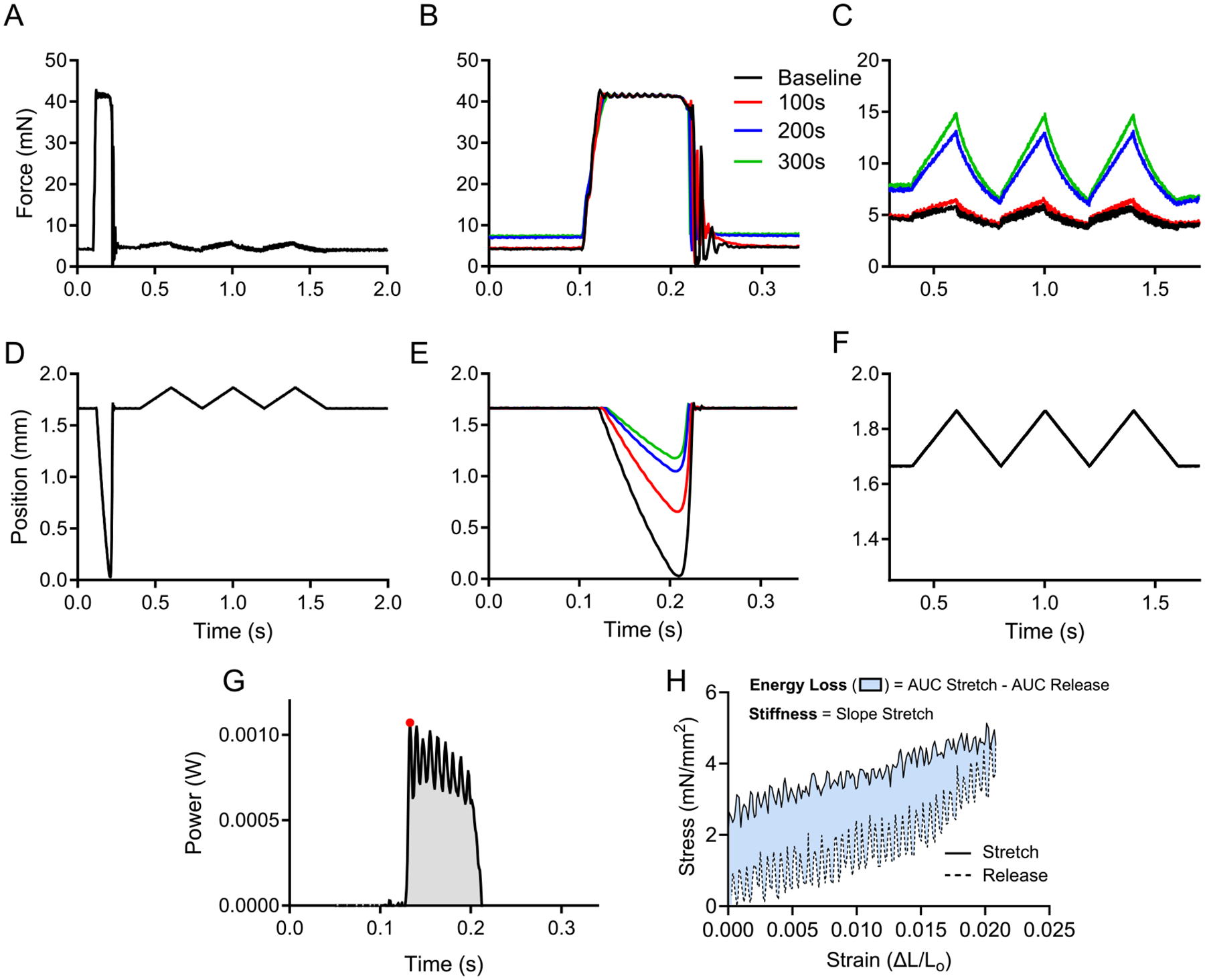
Afterload contractions to study isotonic fatigue. (A, B, C) Force- and (D, E, F) position-time tracings for an afterload contraction implemented in the fatigue protocol. Following activation and shortening, muscles underwent 3 passive stretches to realign sarcomeres and aid in stability of the preparation. (A) and (D) represent one afterload isotonic contraction used to construct the force-velocity and force-power curve in Fig. 2A and B. (B) and (E) are magnified tracings of the active portion of the isotonic contraction, overlaid with tracings from multiple time points during a representative fatigue trial to show changes to velicoty. (C) and (F) are magnified tracings of the passive portion of the isotonic contraction, overlaid with tracings from multiple time points during a representative fatigue trial to show changes to passive mechanical properties of muscle. (G) Representative power-time tracing calculated from force and velocity data. Red dot indiciates measurement for peak power. Integral of positive region used to calculate work performed during the contraction. (H) Example stress-strain relationship to examine passive mechanical properties in response to fatigue.

A bundle from one control animal was dedicated to constructing a force-velocity and force-power curve using afterload contractions [29, 30]. The dual-mode lever arm/force transducer was clamped at various loads ranging from 15 to 70 % of maximal isometric force and the muscle was activated via an electrical stimulation (current: 600 mA; pulse width: 0.25 ms; train duration: 100 ms; frequency: 120 Hz, the lowest frequency that elicited a fused tetanic tracing and reached ~ 90–95 % maximal force in murine diaphragm at 37 °C). Upon activation, muscle length remained constant (isometric phase) until the developed force exceeded the load-clamp, at which point the muscle shortened against the load (isotonic phase; example force- and position time tracing in Fig. 1 A, B, D, E). Instantaneous velocity (mm/s) during muscle activation was calculated as the first derivative of the position-time tracing, while instantaneous power (W/kg) was calculated as the product of instantaneous velocity and corresponding force normalized to bundle weight (N/kg). Peak power was determined as the highest point in the instantaneous power tracing. The integral of instantaneous power during muscle shortening was then used to calculate work (J/kg) performed during shortening (example tracing in Fig. 1G). The force-velocity relationship was plotted and fit using the Hill equation [31]. We calculated the force-time integral from the force-time tracing and delineated discrete isometric and isotonic phases determined by the initiation of shortening in the position-time tracing. Calculations for derivatives and integrals were performed in a computational model using open source libraries [32].

#### c Isotonic Diaphragm Fatigue Protocol

To measure responses to isotonic fatigue, we designed a sequence using repetitive afterload contractions as described above. Stimulations (current: 600 mA; pulse width: 0.25 ms; train duration: 100 ms; frequency: 120 Hz) were repeated 150 times every 2 s (duty cycle: 0.05) yielding a total fatigue protocol time of 5 min. Between each contraction, diaphragm bundles underwent three passive triangular stretch-release cycles (0.2 mm in 0.4 s per each stretch/release phase) from L_o_ (example force- and position-time tracing in Fig. 1A–C, D and F). This stretch cycle commenced 0.2 s after the end of stimulation, allowing for the muscle to reach a recovery steady state before stretch. The first passive stretch-release cycle yielded a triangular ramp that allowed us to examine elastic modulus and hysteresis, and subsequent cycles were employed to minimize the negative impact of active shortening on muscle structure and contractile function [33,34]. For instance, passive stretch-release cycles attenuate instability with repeated activation and shortening in single muscle fibers [33].

Isometric fatigue protocols modify intensity by adjusting the frequency of stimulation, whereas the load during shortening determines the effort of isotonic contractions. In preliminary experiments, we assessed diaphragm fatigue in control animals under loads of 30, 45, and 60 % F_max_. Each load was tested in an individual bundle from an individual control animal. Based on the results of these experiments we proceeded with 45 % F_max_ as the load to study the role of Nox2 on isotonic fatigue.

#### d Fatigue Experiments with Assessment of Recovery

Upon defining the experimental conditions to test active and passive properties of muscle during fatigue in preliminary assesments, we expanded the experimental platform to include assessment of isometric and isotonic properties of muscle during recovery from fatigue. After setting L_o_ and bringing the muscle temperature up to 37 °C, we measured baseline maximal (300 Hz) and submaximal (30 Hz) isometric force (current: 600 mA; pulse width: 0.25 ms; train duration: 300 ms). The muscle rested for 1 min before beginning the afterload fatigue protocol with shortening against a load clamped at 45 % of the maximum force. We then measured isometric force (30 and 300 Hz) and power (afterload contraction at 45 % maximal ref force) at 10 s (‘immediate post’), 5 min, 30 min and 60 min after the conclusion of the 5 min fatiguing protocol to observe the recovery profile. Bundle length and weight were then measured for normalization (fatigue trial and recovery schematic in Fig. 4).

#### e. Data Analysis

Power and work during fatiguing contractions were compared between groups in absolute (W/kg and J/kg, respectively) and relative (% baseline) values. Total work was calculated as sum of isotonic work performed in each contraction of the protocol. Prolonged low frequency force depression (PLFFD) was calculated as the ratio of submaximal isometric force (30 Hz) to maximal isometric force (300 Hz) normalized to the pre-fatigue ratio. Recovery kinetics for power, work, and maximal isometric force were calculated individually for each animal. Power, work, or force as a percentage of baseline measurements immediately following fatiguing contractions were subtracted from recordings at 5-, 30-, and 60-min post-fatigue. Based on visual inspection of the data, a nonlinear regression saturation binding model [Y = (B_max_*X)/(K_d_ + X), GraphPad Prism v.9.1] was used to establish recovery kinetics with measurements taken immediately post fatigue serving as ‘time zero’. In this model, B_max_ represents maximum recovered power, work, or force (P_max_ W_max_ or F_max_) and K_d_ is the time to 50 % recovery (T_50_).

Passive mechanical properties were assessed in the first passive stretch-release cycle (region of interest 0.4–0.8 s) following contractions 1, 15, 30, 60, 90, and 150 and during recovery (0, 5, 30, 60 min after the last fatiguing contraction). We assessed normalized baseline tension, energy loss, and stiffness. Baseline tension was measured just before muscle stimulation. Graphing the stress-strain relationship during the first triangular stretch/release cycle allowed us to calculate energy loss by subtracting the total area under the curve (AUC) during the stretch phase by the total AUC during the release phase (Energy loss = AUC stretch – release) (example stress-strain tracing Fig. 1H). Stiffness was obtained by calculating the slope of the stress-strain relationship.

### 2.3. Statistics

Data in text are presented as mean ± SD. We performed tests for normality (Shapiro-Wilk) and equal variance (Brown-Forsythe) and transformed data that did not pass these tests before comparing skmNox2 and skmNox2^KO^ groups via Student’s unpaired *t*-tests (control vs. Nox2 KO) or repeated measures two-way analysis of variance (ANOVA) (main effects: control vs. Nox2 KO; time during during recovery). We proceeded with post-hoc (Bonferroni *t*-test) analysis if the p-value from the interaction of main effects <0.05.

## 3. Results

### 3.1. Isotonic load and fatigue

**Fig. 2.**
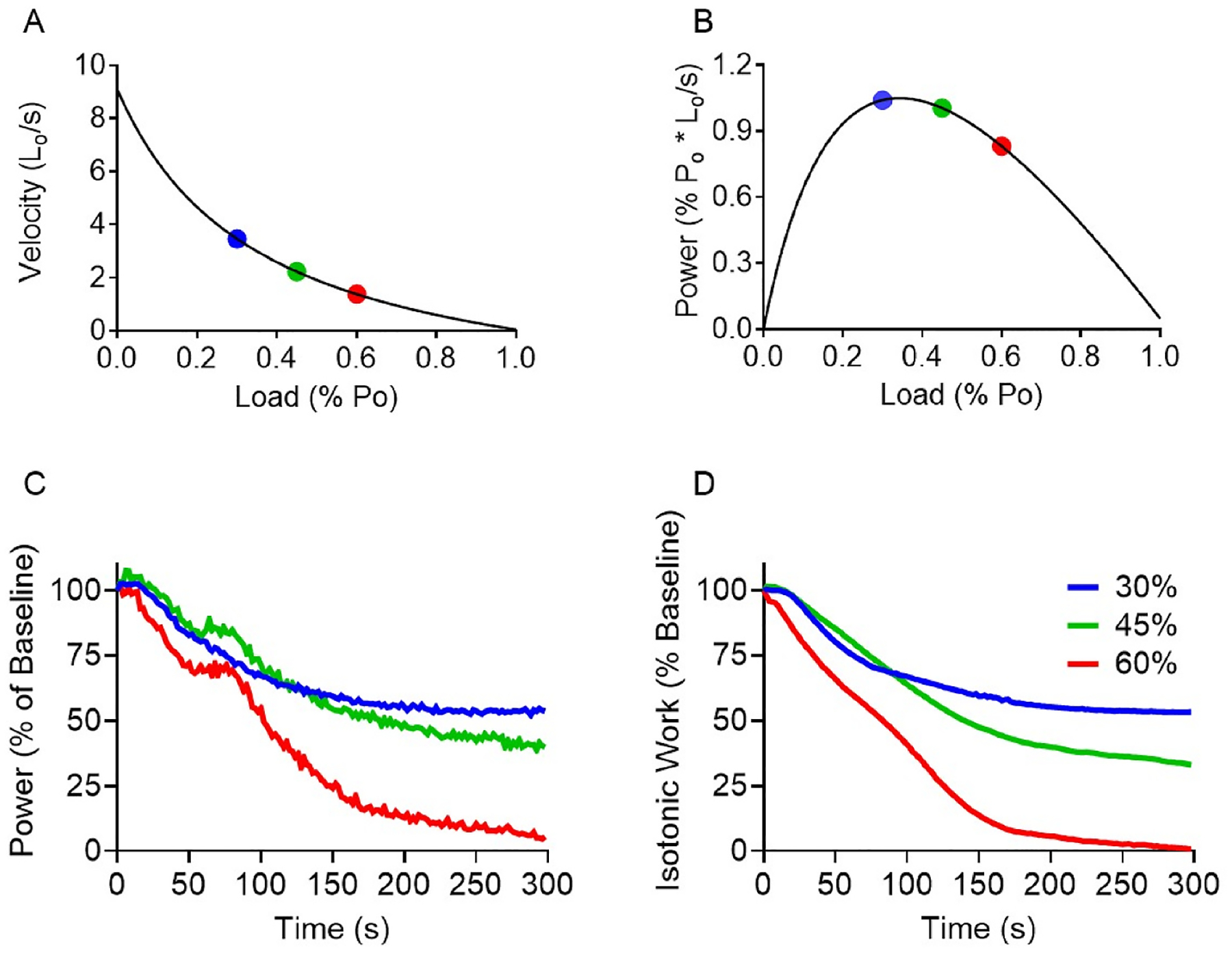
Shortening load during isotonic fatigue. Example force-velocity (A) and force-power (B) curves indicating shortening loads (open shapes) tested in preliminary fatigue trials in (C and D). Peak power occurs at ~30 % F_max_ and has previously been used as the shortening load in isotonic fatigue experiments. The more pronounced decay in power (C) and isotonic work (D) indicate that fatigue occurs to a greater extent when the shortening load is set at 45 % F_max_, and to a severe extent at 60 % F_max_. Presented data in (C) and (D) are from three different diaphragm bundles isolated from three different control animals.

Traditional isometric fatigue protocols adjust intensity by varying stimulation frequency, whereas load during shortening dictates intensity in isotonic contractions. Our objective in preliminary experiments testing fatigue against different percentages of maximal force was to determine the optimal afterload for subsequent experiments, roughly defined as load that induced >50 % decline in power output within ~5 min. Based on studies in humans [35], we considered that >50 % decline in muscle function in ~5 min would be consistent with contractions performed near or within the severe exercise intensity domain. With the load set at 30 % F_max_, power did not fall below 50 % baseline within 5 min. We increased the load to 60 % F_max_ in subsequent preparations, which caused a rapid and excessive decline to ~10–20 % baseline power. As an intermediate, we tested 45 % of the maximum force and found that this load produced consistent fatigue to 30–35 % of baseline power (65–70 % decline in power) at the end of the protocol (Fig. 2C).

**Fig. 3.**
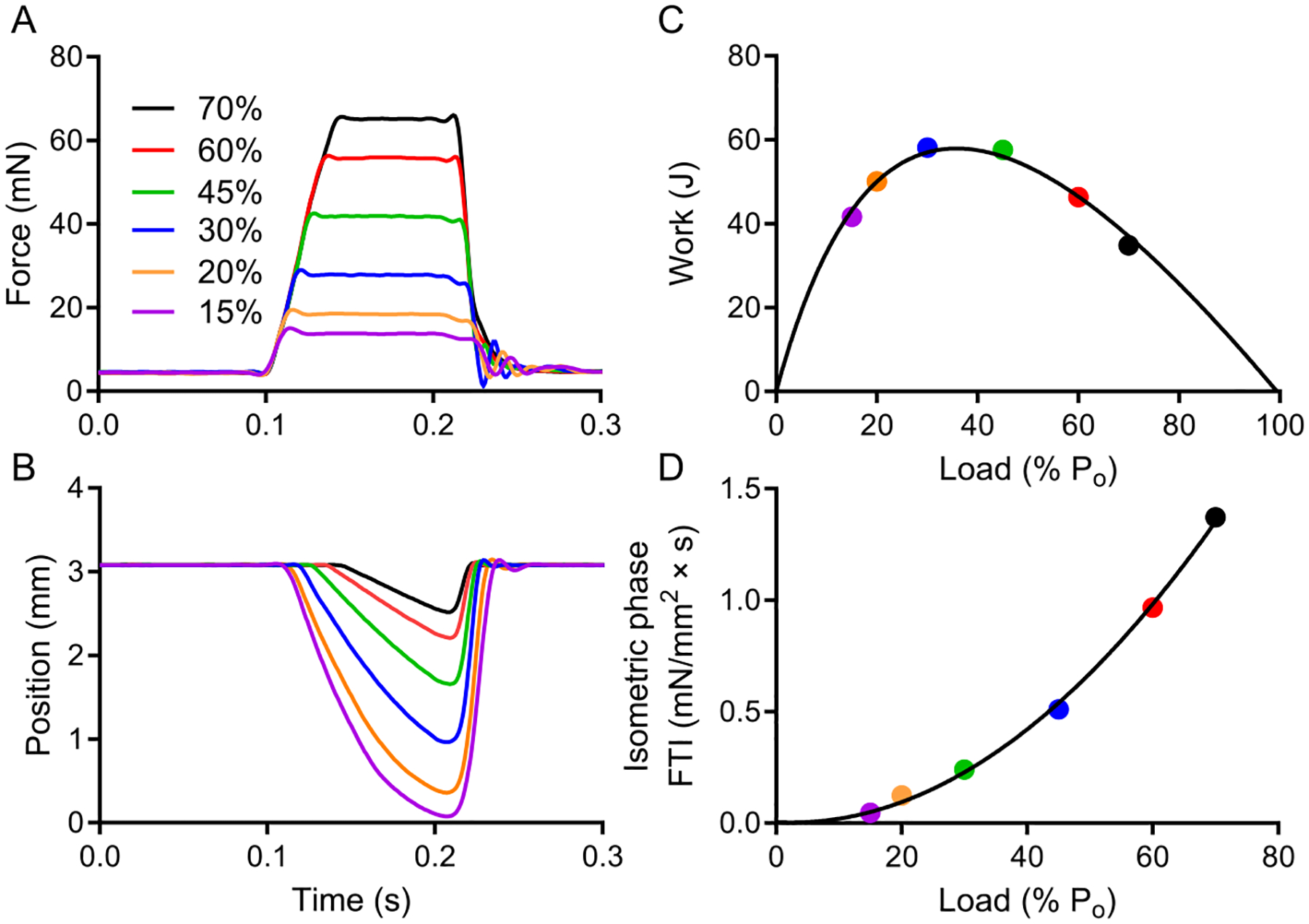
Mechanical Properties under various loads during muscle shortening. Force-time (A) and position-time (B) tracings of muscle activation and shortening under various loads as a percentage of Fmax. The energy associated with an isotonic contraction can be considered as the energetic cost of isometric force and the work performed during shortening. (C) Work performed during shortening resembles the force-power curve from Fig. 2B; however, the force-time integral of the isometric phase of muscle contraction (D) increases exponentially with shortening load. These relationships indicate the isometric phase heavily contributes to energetic costs during afterload isotonic contractions.

The isotonic fatigue profiles shown in Fig. 2C were surprising to us as previous studies have shown that isotonic contractions against ~30 % maximal force elicit the highest metabolic demand [36–40]. To understand the fatigue profiles with afterload isotonic contractions, we next assessed the mechanical properties of several afterload contractions in a healthy, unfatigued muscle. Force-time and position-time tracings (Fig. 3A and B) were used to calculate work performed during isotonic shortening (Fig. 3C) and the force-time integral during the isometric phase (Fig. 3 D). This approach permits assessment of discrete isometric and isotonic components of an afterload contraction. Work performed during shortening under various loads followed a similar tracing to the force-power relationship with a plateau around 30–45 % F_max_. However, the force-time integral during the isometric phase of contraction increased exponentially with increasing load during shortening.

### 3.2. Effects of Nox2 on isotonic fatigue

**Fig. 4.**
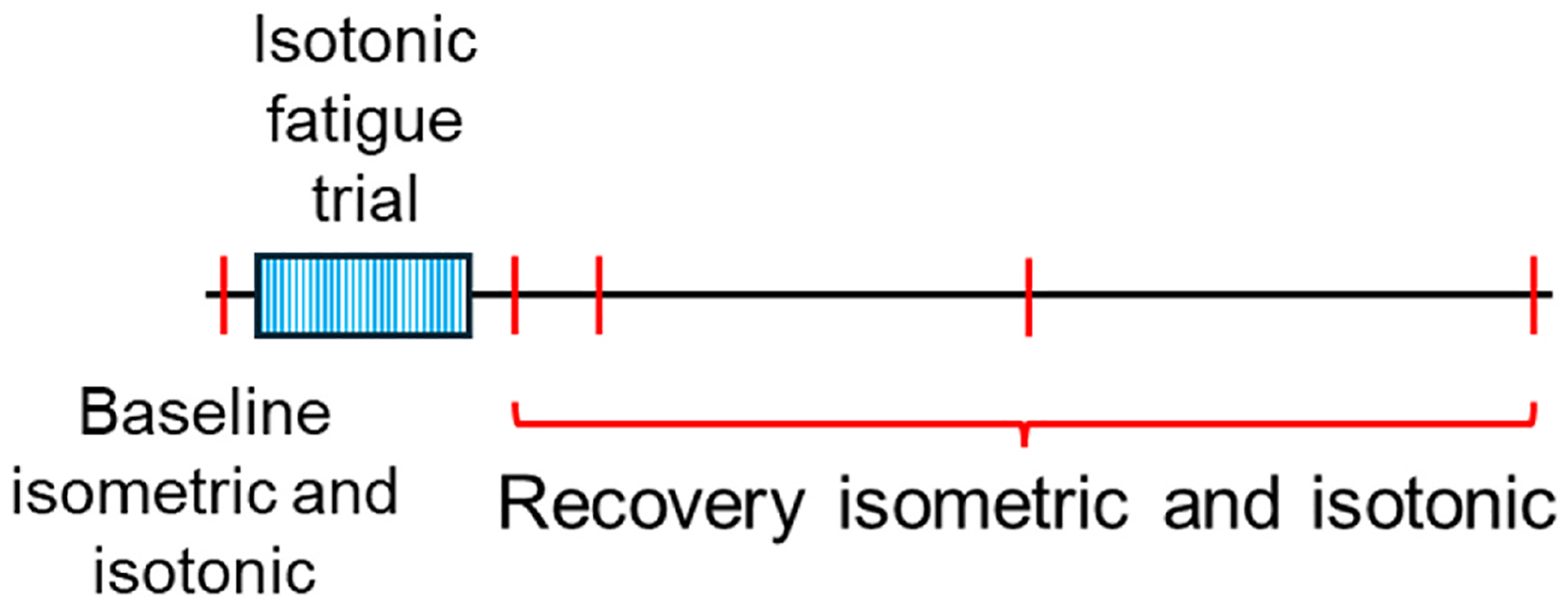
Schematic of isotonic fatigue protocol with assessment of recovery. Isolated murine diaphragm bundles were subjected to repetitive isotonic contractions against a fix shortening load as a percentage of maximum force, indicated by the box with repetitive vertical blue lines. For testing the role of Nox2 on fatigue, this load was selected as 45 % of the maximum isometric force based on preliminary results in Fig. 2. Prior to the fatigue protocol, bundles were subjected to baseline assessments of maximal (300 Hz) and submaximal (30 Hz) isometric force, and an isotonic contraction against 45 % of maxmimum isometric force. These measurements were then repeated 10 s, 5 min, 30 min, and 60 min following the fatigue protocol to assess recoveryfrom fatigue. Red vertical lines indicate measurement of baseline and recovery parameters.

**Fig. 5.**
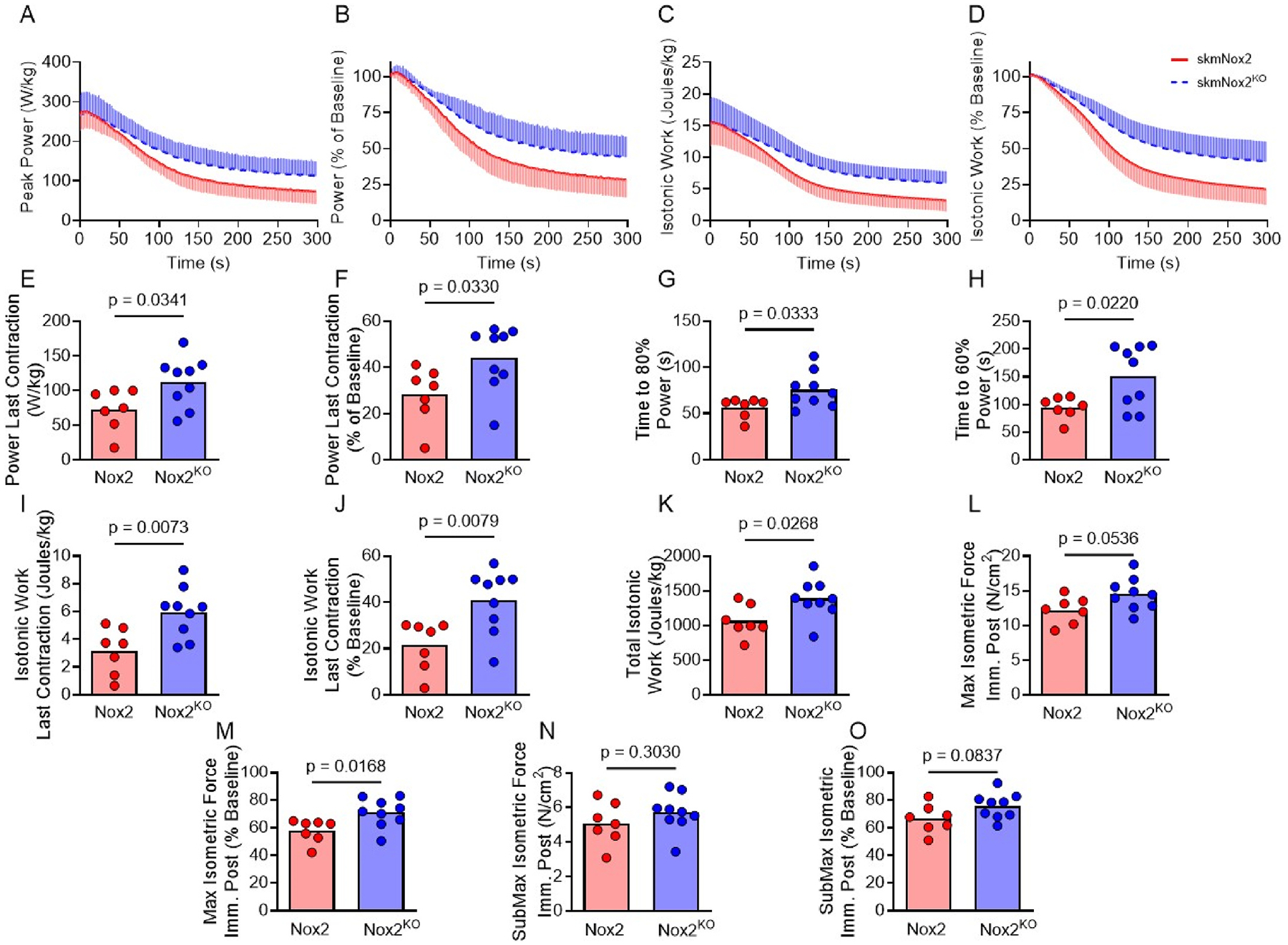
Knockout of skeletal muscle Nox2 improves active contractile properties during isotonic fatiguing contractions. Changes in power (A, B) and isotonic work (C, D) through the fatigue protocol. Knockout of Nox2 (blue dots; dashed blue line) resulted in greater power (E, F) and isotonic work (I, J) at the end of the fatigue protocol, a slower decline in power (G, H) and more total work performed (K) during isotonic fatigue. Maximal isometric force measured immediately post fatigue protocol was higher with the knockout of Nox2 (L and M) but not submaximal force (N and O). Statistical analyses by unpaired *t*-test.

We then applied the new fatiguing protocol (schematic portrayed in Fig. 4) to diaphragm bundles from skmNox2 and skmNox2^KO^ animals, comparing absolute (W/kg, Fig. 5 A) and relative (% Baseline, Fig. 5 B) power. Power dropped to ~30 % baseline values at the end of the 5-min protocol in control animals, but only 45 % in skmNox2^KO^ animals (*p* = 0.0330) (Fig. 5B–F). Control animals fatigued at a faster rate, reaching 80 % and 60 % of baseline power faster than skmNox2^KO^ animals (Fig. 5G and H). Knockout of skmNox2^KO^ also resulted in greater work performed in the final contraction of the fatigue protocol (Fig. 5 I; p = 0.0073, ~40 % baseline compared to ~20 % baseline of controls (Fig. 5 J; p = 0.0079), and greater total isotonic work performed during the fatigue protocol compared to controls (Fig. 5 K, p = 0.0268). Maximal isometric specific force (300 Hz) decreased to a lesser extent in skmNox2^KO^ animals (70 % baseline) compared to controls (57 % of baseline) (*p* = 0.017) (Fig. 5 M). Submaximal isometric force (30 Hz) fell to 66 and 75 % baseline in control and skmNox2^KO^ and groups, respectively (*p* = 0.084) (Fig. 5 O).

### 3.3. Effects of isotonic fatigue on passive mechanical properties of muscle

**Fig. 6.**
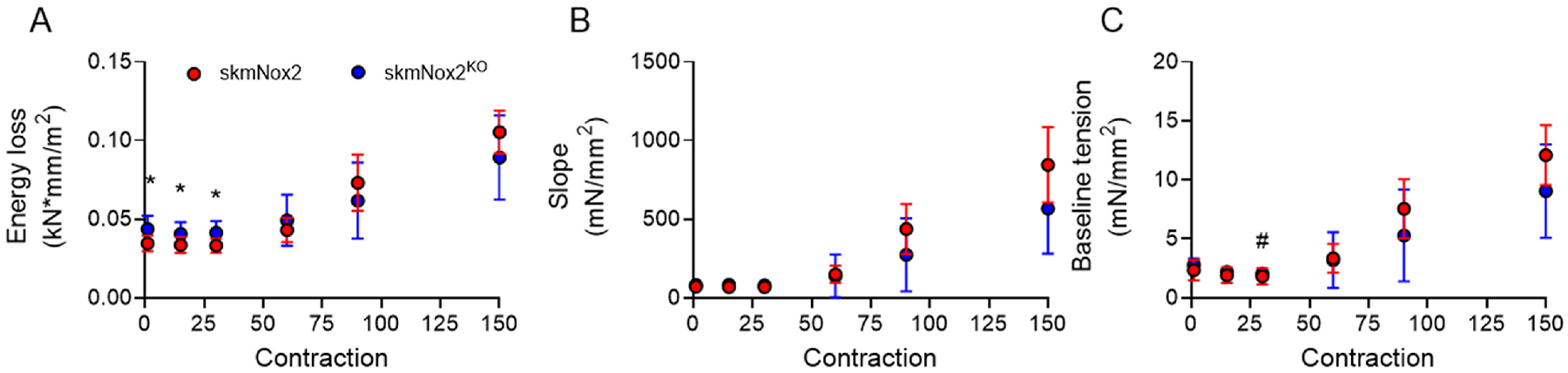
Impact of repetitive isotonic contractions on passive mechanical properties. Diaphragm passive mechanical properties of skmNox2 (red) and skmNo2^KO^ (blue) mice between series of fatiguing contractions. Energy loss (A), total area under the curve (AUC) during stretch minus total AUC during release; stiffness (B), slope of the stress-strain relationship; normalized baseline tension (C), baseline passive force divided by CSA. Statistical analyses by RM Two-way ANOVA with Bonferroni post-hoc test. *p < 0.05 significantly different between groups; # significantly different from Contraction 0.

Repeated isotonic contractions caused an increase in diaphragm energy loss and stiffness (slope of the stress-strain relationship (contraction effect of p < 0.001; Fig. 6A and B)). Baseline tension initially dropped (Initial vs. Contraction 30, 2.61 ± 0.71 vs. 1.97 ± 0.52; *p* = 0.032) before rapidly increasing like other passive properties (Initial vs. Contraction 150, 2.61 ± 0.71 vs. 10.38 ± 3.66; *p* < 0.001) (Fig. 6C). The skmNox2 and skmNo2^KO^ mice presented similar passive mechanical properties during fatigue (no effect of Nox2). However, our analysis indicated a *Nox2 vs*. *contraction* interaction effect of *p* ≤ 0.01 in all passive measurements. Subsequent multiple comparison testing revealed that skmNo2^KO^ mice displayed greater energy loss at baseline and early fatigue (Fig. 6 A).

### 3.4. Recovery following isotonic contractions

**Fig. 7.**
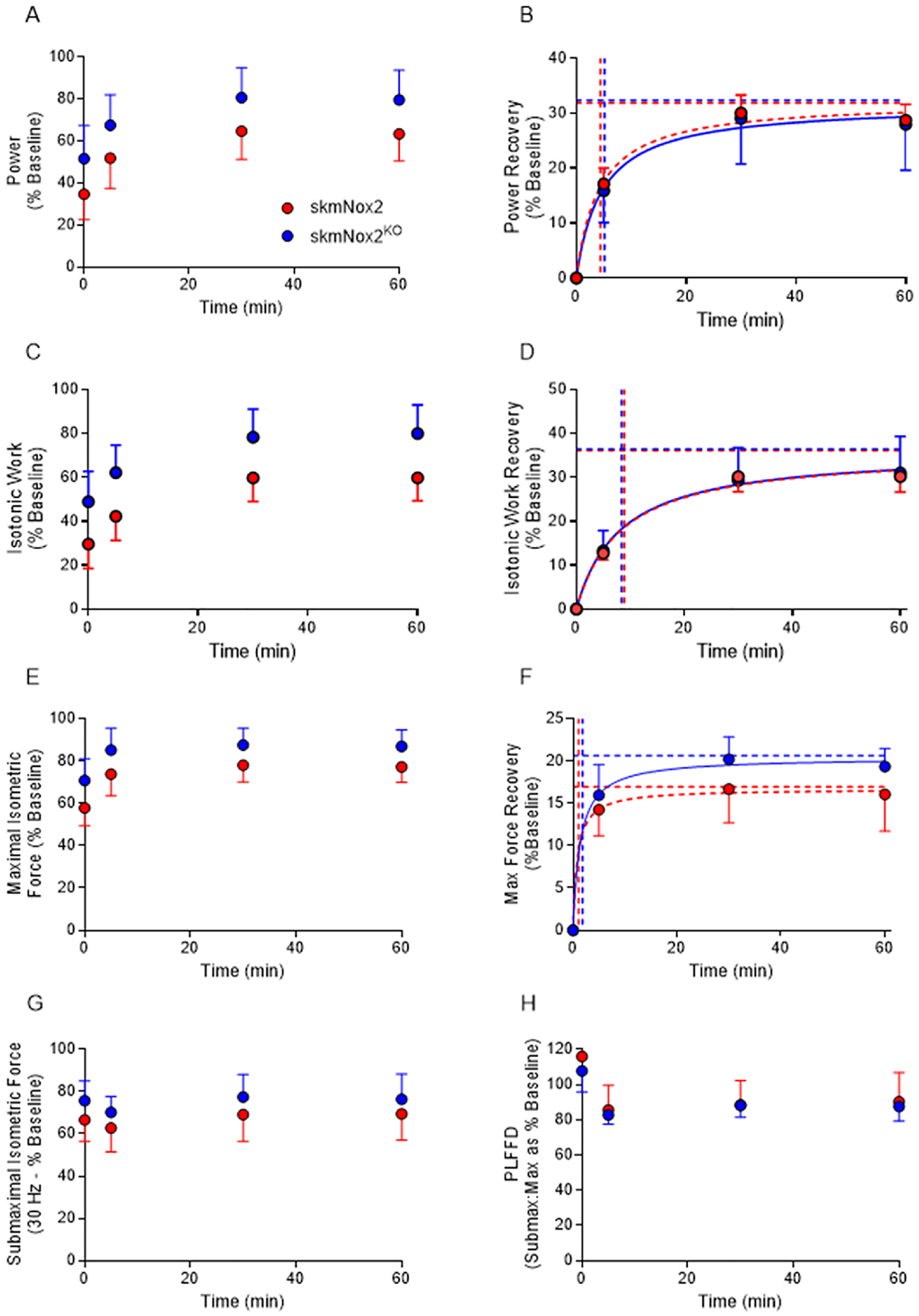
Post-fatigue recovery of power, maximal isometric force, and submaximal isometric force. (A) Power and isotonic work (C) fell to a greater extent in control animals post-fatigue and remained lower throughout 60 min of recovery. (B) Power and isotonic work (D) recovery kinetics were not different between skmNox2^KO^ (blue) and skmNox2 (red) animals. Horizontal dashed lines signify maximal recovered power (P_max_) or maximal isotonic work, vertical dashed lines signify T_50_, time to 50 % recovery of maximum. (E) Maximal specific force dropped to a greater extent in control animals and remained lower throughout 60 min of recovery. (F) Maximal isometric force recovered to a greater extent in control animals but did not reach significance. Horizontal dashed lines signify maximal recovered isometric force (F_max_). Vertical lines signify T_50_. (G) Submaximal force decreased in the first 5 min of recovery and remained depressed through the 60 min recovery period. (H) Prolonged low frequency force depression (PLFDD), calculated as the ratio of submaximal to maximal force divided by the pre-fatigue ratio, remained depressed for the 60 min recovery period.

In general, the ~15 % difference in power between groups that was apparent following fatiguing contractions persisted throughout recovery (Fig. 7A and B). By 30 min, power had recovered to 83 % baseline and plateaued in the skmNox2^KO^ group, but only to 65 % in controls. Recovery kinetics were not different, with both groups recovering ~35 % of power lost after the fatigue trial (skmNox2 vs. skmNox2^KO^, P_max_: 32.37 ± 3.37 vs. 31.89 ± 10.02 % Baseline, *p* = 0.9043; T_50_: 4.42 ± 1.85 vs. 5.14 ± 2.87 s, *p* = 0.5781; comparisons by unpaired student’s *t*-test). Similarly to power, the ~20 % difference in isotonic work between groups immediately after fatigue persisted throughout the recovery phase. Also, by the 30 min mark, isotonic work recovered to 80 % baseline in skmNox2^KO^ and only to 60 % in controls. Recovery kinetics of isotonic work were also not different between groups (skmNox2 vs. skmNox2^KO^, maximum: 36.09 ± 4.50 vs. 36.45 ± 10.57 % Baseline, *p* = 0.9332; T_50_: 8.43 ± 1.23 vs. 8.91 ± 3.62 s, *p* = 0.7439; comparisons by unpaired student’s *t*-test).

Recovery of F_max_ in the skmNox2^KO^ group was largely complete within the first 5 min of recovery, whereas the control group continued to improve through 30 min. As such, control animals exhibited a larger F_max_ that approached significance (skmNox2 vs. skmNox2^KO^: 20.66 ± 2.66 vs. 16.98 ± 4.56 % Baseline, *p* = 0.079; comparisons by unpaired student’s *t*-test) but no difference in T_50_ (1.85 ± 2.56 vs. 1.09 ± 1.51 s, *p* = 0.468; comparisons by unpaired student’s *t*-test) (Fig. 7E and F).

Submaximal force continued to gradually decline through the first 5 min of recovery (Fig. 7 G) and remained depressed 30- and 60-min post fatigue in both groups, indicative of PLFFD. The ratio of submaximal to maximal force dropped and remained depressed up to 60 min after the fatigue protocol, with no effect of Nox2^KO^ (Fig. 7H).

**Fig. 8.**
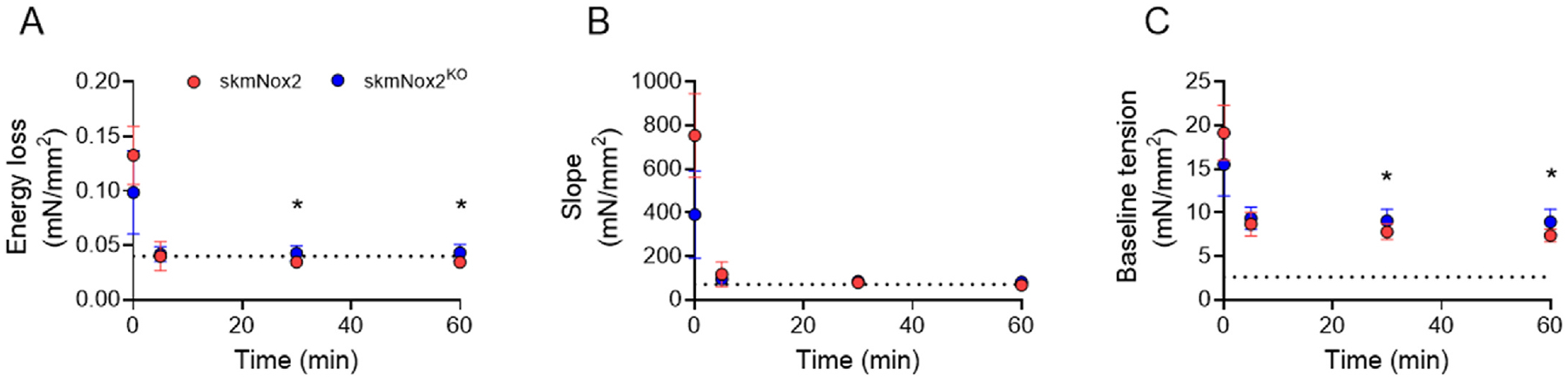
Recovery of passive mechanical properties. Following repetitive isotonic contractions, energy loss (A) and stiffness (B) returned to baseline values within 5 min. Baseline tension (C) decreased, but remained elevated from pre-fatigue values for the 60 min observed. Dashed lines represent pre-fatigue values from contraction 0 in Fig. 6. Statistical analyses by RM Two-way ANOVA with Bonferroni post-hoc test. *p < 0.05 significantly different between groups.

Energy loss and stiffness recovered to baseline values after 5 min of recovery in both groups (recovery time effect p < 0.001) (Fig. 8A and B). Passive tension declined after 5 min, (recovery time effect p < 0.001) but remained elevated from baseline values (Baseline vs. 60 min post, baseline tension: 2.61 ± 0.71 vs. 60 min post: 8.25 ± 1.41; *p* < 0.001) (Fig. 8C).

## 4. Discussion

Our study introduces a physiologically relevant approach to examine active and passive mechanics during isotonic fatigue in isolated muscles *ex vivo*. The protocol we devised and resulting data reveal novel features of isometric load and isotonic work during afterload isotonic contractions that help reconcile apparent discrepancies among data from muscle power, metabolic rate, and fatigue *ex vivo* and results from exercising humans and animals. We also observed that isotonic fatiguing contractions lead to a delayed and exponential increase in baseline tension, stiffness, and energy loss (viscosity) during stretch-release cycles. Applying this novel approach, we found that knockout of skeletal muscle Nox2 slowed the decline in muscle peak power during repetitive isotonic contractions against 45 % of maximal isometric force. However, Nox2 knockout did not affect changes to passive mechanical properties, the development of PLFFD or the recovery kinetics of maximal isometric force and power.

### 4.1. Isotonic Fatigue and Mechanical Load

At its principal form, fatigue describes an inability of muscle to maintain force or power output. Muscles contract and produce force through cyclic interactions between actin and myosin at the expense of ATP. If the energetic demand associated with an intensity is below a particular threshold, ATP re-synthesis is well maintained by oxidative phosphorylation from the mitochondria once a metabolic steady state is reached and contractile activity can be sustained for extended periods (>hours). As soon as exercise intensity and ATP demand exceeds the rate of ATP synthesis from oxidative phosphorylation, there is increased reliance on finite metabolism (glycolysis, creatine kinase, adenylate kinase) [41]. With increased reliance on non-oxidative metabolism, loss of mechanical power will occur within seconds to minutes depending on the intensity as metabolites associated with ATP hydrolysis accumulate and interfere with force and power production [41]. The determinants of fatigue are thus largely based on a balance between the rate of ATP consumption and the ability of the mitochondria to maintain metabolic equilibrium.

Any alteration to mechanics of contraction during exercise will change the energetic demand and thus the rate at which fatigue develops. Our implementation of isotonic contractions over the traditionally used isometric contractions adds physiological relevance and ecological validity to our *ex vivo* experiments. In our preliminary experiments, the mechanical load under which the muscle shortens was manipulated to vary intensity. We initially measured fatigue while shortening against 30 % F_max_, which corresponds to peak power based on the force-power curve [31,42] (Fig. 2 B). Previous studies investigating isotonic fatigue used this load with the rationale that peak power would elicit the fastest rate of fatigue, but no measurements on other loads were provided to support this claim [38–40]. Fatiguing contractions at 30 % F_max_ plateaued above 50 % of baseline power at the end of the 5-min period. It was at higher loads of 45 % F_max_ that power declined to a greater extent, and to a severe extent at 60 % F_max_.

Muscle shortening is more metabolically demanding than an isometric contraction [36,43]. This is due to the higher ATPase activity of cross bridge cycling as the muscle shortens under load. This concept relates back to the Fenn effect, which is the observation that muscle produces heat dependent on the work that it performs [36]. Prof. Jack Rall proposed that the Fenn effect could be quantified by a simple equation where the energy associated with a muscle contraction is equal to the energetic cost of isometric force plus the work done by the muscle [44]. However, Rall’s proposal, based on experiments performed with isotonic release contractions, only considers the force *during* the shortening phase. It did not account for the force during the isometric phase that preceded the onset of shortening, which is equal across loads when working with isotonic release method. Both the isometric force and work performed during shortening are influenced by the shortening load in afterload contractions, but as a bell-shaped curve for the work performed during shortening (Fig. 3C) and as an exponential curve for the isometric force (Fig. 3 D).

While it is well established that muscle shortening incurs a greater metabolic demand than fixed-length contractions [36,43,44], the effect of load during shortening on fatigue has not been fully explored. From a conventional perspective, our observation of a faster rate of fatigue with a higher load during shortening seems appropriate, and perhaps even obvious. However, this initially conflicts with known biophysical aspects of cross bridge kinetics and muscle shortening. Pioneering experiments of A.V. Hill indicate that peak power and heat production occur when shortening against ~30 % F_max_ [31]. This is further supported from the work of Prof. Gary Sieck, who determined that the relationship between ATPase activity and shortening under variable loads follows the same relationship as the force-power curve, with a peak in ATPase activity occurring when shortening under 30 % F_max_ [37]. With this understanding, we and others [38–40] predicted that shortening against this load would elicit the highest metabolic demand and the fastest rate of fatigue, but this was not the case. To clarify this disconnect, we revisited Rall’s argument about the Fenn effect [44] and expanded the concept by examining afterload contractions in discrete isotonic and isometric phases.

The amount of work performed during the isotonic phase of an afterload contraction can be quantified as the instantaneous power-time integral. The relationship between shortening load and work in an unfatigued muscle is similar to the force-power curve, with a peak at approximately 30 % F_max_ (Fig. 3C). However, this analysis ignores the energetics of the isometric phase preceding the onset of shortening. Theoretically, (external) work cannot be calculated during an isometric contraction. Ortega et al. described a linear positive relationship between the energetic cost of contraction and the force-time integral (FTI) of an isometric contraction [43], which suggests that the FTI is an indicator of internal work and energetics of an isometrically contracting muscle. Accordingly, the isometric FTI predicts the rate of isometric fatigue development [45]. Thus, we determined differences in ‘energetics’ of the isometric phase, quantified by FTI, under different mechanical loads tested here. We fit a quadratic function to the relationship between isometric phase FTI and load (Y = 0.004451 – 0.001398X + 0.0002950X^2^) (Fig. 3D). The concave-upward curve indicates a disproportionate increase in FTI at higher loads, suggesting that the energetic cost of isometric contraction rises non-linearly with increasing load.

The apparent disconnect between our results and traditional biophysical concepts likely comes from differences in the experimental conditions for muscle shortening (see Fig. S1 for comparative force- and position-time tracings of afterload and isotonic release contractions). Isovelocity or isotonic release contractions are commonly used to study the force-velocity relationship, cross bridge cycling, and efficiency of muscle contraction. With these approaches, isometric force peaks and plateaus before a sudden submaximal load clamp when shortening occurs [37,46]. Thus, any changes in ATPase activity at various submaximal loads can be attributed to differences in the isotonic phase because the isometric phase is identical for each load. Contrarily, the load during an afterload contraction, employed here, impacts both the isometric and isotonic phase. To investigate the fundamental biophysics of muscle contraction, isotonic release protocols are typically more appropriate. However, afterload contractions are more akin to how muscles contract and produce power *in vivo* and thus more useful for assessment of muscle contraction for translation to *in vivo* function and studies of isotonic fatigue.

The translational relevance of the approach we developed for isotonic fatigue *in vitro* has been validated recently with assessment of muscle function *in situ* [47]. The fatigue protocol and initial observations described in the current study, which have been available in preliminary form since 2022 [48], laid the foundation for Palzkill et al. [47] to implement a 6-min fatigue test that captured differences in muscle function caused by peripheral artery disease (PAD) myopathy and therapeutic responses that is more akin to clinical assessments of muscle function in patients with PAD [49].

It is worth highlighting that the ‘optimal’ load for the isotonic fatigue protocol will likely vary with conditions under investigation. Other muscles or disease conditions might require a lower load to avoid cessation of isotonic contractions within the 5-min window. The 45 % F_max_ load proved valid for the purposes of our study and later translated well to an *in vivo* preparation [47].

### 4.2. Nox2 and skeletal muscle fatigue

Knockout of Nox2 from skeletal muscle improved power production at the end of the fatigue trial (in W/kg: 112.80 ± 35.99 vs. 72.98 ± 30.20 in control animals, Fig. 5E). Fatigue also developed at a slower rate with the knockout of Nox2, exemplified by a slower time to 80 % and 60 % baseline power compared to controls. While control animals fatigued to a greater extent, recovery kinetics were not different between control and Nox2^KO^ muscle bundles. Maximal isometric force largely recovered within 5 min, while power continued to recover over 30 min.

It is difficult to delineate the mechanisms behind improved power with the knockout of Nox2 with the measurements at hand. An improvement in fatigue, first and foremost, suggests an improved ability to maintain metabolic equilibrium during repetitive muscle activation, perhaps due to enhanced mitochondrial function. However, we observed no fiber type shifts or changes in mitochondrial content and function with the knockout of Nox2 in our previous experiments [25]. Using the same mouse model, this included assessment of respiration in permeabilized diaphragm bundles, and assessment of mitochondrial oxidative phosphorylation complexes via Western blot. The impact of knocking out an enzyme can be considered in the context of the effect on the substrates and products of its reaction. In the case of Nox2, NAD(P)H and O_2_ are consumed, resulting in the formation of superoxide and NAD (P)^+^. Excess NAD(P)H and O_2_ spared from consumption by Nox2 could result in better maintenance of metabolic equilibrium. Alternatively, it is possible that ROS produced from Nox2 directly impairs mitochondrial function through oxidation of mitochondrial proteins that increases with fatiguing contractions [50,51]. Furthermore, ROS from Nox2 may directly contribute to the decrease in shortening velocity that occurs with fatiguing contractions through oxidation of myofibrillar proteins that impair cross bridge cycling. Impaired excitation-contraction also contributes to impaired contractile function with fatigue, and the knockout of Nox2 may mitigate this via its proximity to the endoplasmic reticulum.

It is well established that ROS contribute to fatigue, as evidenced by increased performance (*in vitro* and *in vivo*) following the administration of N-acetylcysteine (NAC). However, the exact source(s) of ROS that contribute to fatigue have not been elucidated. Evidence over the past years has pointed to Nox2 as the predominant source of ROS during contractions, and several previous studies have indicated that pharmacological blockade or genetic deletion of Nox2 prevents the contraction-mediated increase in cytosolic ROS [20,22,52]. However, two previous studies investigating the role of Nox2 on isometric fatigue both found no effect from Nox2 genetic deletion or pharmacological inhibition [23, 53]. The first of these studies assessed isometric fatigue in isolated murine diaphragm strips with a genetic knockout of Nox2. Muscles were stimulated at 40 Hz for 5 min with a train duration of 400 ms every 2 s (duty cycle: 0.2). Force dropped to ~25 % baseline at the end of the protocol and was not different between knockout and control groups [23]. The second study assessed fatigue in murine FDB fibers treated with NAC or the Nox2 inhibitor gp91ds-tat. Fibers underwent 60 isometric stimulations (train duration 150 ms) delivered at 70 Hz every 1 s (duty cycle: 0.15). Force dropped to ~30–40 % baseline and was not effected by Nox2 inhibition or NAC treatment [53]. The authors suggested that the lack of protection from NAC stemmed from the use of a ‘high intensity fatiguing stimulation protocol, where severe fatigue occurred within 1 min’, whereas ‘a clear positive effect (of ROS-neutralizing agents) on endurance is observed with submaximal contractions’ [53].

A few key differences can be noted between the two previously mentioned studies and the one described here: 1) stimulation frequency; 2) duty cycle; and 3) the type of contraction. As discussed earlier, all three factors contribute to the rate and mechanisms of fatigue development. While the stimulation frequency used in our experiments was higher than both previous studies (120 Hz), the duty cycle was also shorter (0.05), permitting more rest in between contractions. Furthermore, this stimulation frequency was selected to elicit fused tetanus to aid in the calculation of shortening velocity during muscle shortening, whereas the afterload during shortening was altered to modify intensity. This is contrary to isometric protocols, where the stimulation frequency is selected to determine the amount of force produced and, thus, intensity. The tension-time index (TTI) accounts for both stimulation frequency (i.e. intensity) and duty cycle and is a major determinant of fatigue [45]. For the purpose of comparing experimental approaches, we subjected a control diaphragm bundle to 10 s of conditions of the fatigue protocols described above, interspersed by 5 min between each protocol. The intent was not to induce fatigue, but to calculate and compare the TTI from each protocol. TTI in the previous studies’ isometric protocols was higher (0.034 [23] and 0.099 [53]) than the protocol implemented here (0.024), owing to a longer duty cycle (Fig. S2). In the initial description of the TTI, Klawitter and Clanton [45] indicate that muscle struggles to maintain active force when TTI values are set above 0.08. Arguably the most substantial difference in experimental design between the aforementioned and current study is the use of isotonic contractions herein. In the case of the study by Loehr et al., a 40 Hz stimulation used to fatigue muscle elicits approximately 50 % of maximal isometric force, estimated from the provided force-frequency curve [23]. A 70 % decrease in force is observed over the 5 min fatigue protocol, and no differences are observed between control and Nox2 KO muscle bundles. In the case of the protocol implemented here, muscles were tasked with shortening against 45 % of maximal isometric load, and control bundles decreased power production by ~70 % over the same time period. These protocols share similar models (Nox2 KO in murine diaphragm bundles), intensities (40 Hz isometric stimulation vs. shortening against 45 % of maximal isometric force), time periods (5 min) and decrements in control muscle bundles (70 % decline in 40 Hz elicted isometric force vs. ~70 % decline in power). It is only with the use of physiological isotonic contractions that the effects of Nox2 on fatigue become apparent. Implementing cross bridge cycling and muscle shortening may reveal mechanisms of fatigue and effects of ROS that are not observable with fixed length contractions.

An important qualifier to the results of this study is that Nox2 may not be the only source of ROS during fatiguing contractions. Knockout of Nox2 improves fatigue and this was the only source of skeletal muscle ROS investigated in this study, but additional sources may still be involved. Importantly, compensatory changes to other ROS sources may occur with the knockout of Nox2, as we and others have observed [25, 54].

### 4.3. Mechanisms of increased energy loss and altered stiffness in isotonic fatigue

Energy loss was constant early in the isotonic fatigue protocol but increased during late fatigue (>75 contractions) in both groups. An increase in energy loss, indicative of increased muscle viscosity following repeated maximum isometric contractions, agrees with previous *in vivo* measurements using shear-wave spectroscopy sequence [3]. We also observed an initial decrease, then a progressive increase in baseline force, suggestive of increased muscle stiffness toward the end of the fatiguing protocol. There have been several previous reports of increased [3–5,55] and one observation of decreased stiffness [56] with fatiguing contractions. The response we observed here indicates two phases of passive mechanics to repeated contractions, where later events cause passive force to greatly increase. This also suggests that the type of contraction and degree of fatigue are important variables when investigating responses of passive muscle stiffness to repeated contractions.

The reversion of energy loss and stiffness to pre-fatigue values within 5 min of recovery indicates acute and quickly reversible mechanisms to their rapid increases during late fatigue. Acute alterations to sarcomeric proteins are a primary candidate for the increased stiffness and viscosity during late fatigue reported here. Titin contributes to overall muscle stiffness and viscoelasticity through the PEVK domain [57] or through interactions with other sarcomeric proteins [58]. Titin redox state and charge also interfere with muscle stiffness [59,60]. Additionally, binding of P_i_ to titin influence titin stiffness [61,62] and the location of P_i_ binding on titin dictates the mechanical change, towards higher or lower stiffness [5,63]. Therefore, changes in titin redox balance and/or phosphorylation levels by increased P_i_ or activation/deactivation of kinases and phosphatases could be contributing to the alterations in the viscoelastic properties of muscle in late isotonic fatigue. Actin-myosin cross-bridge formation also influences muscle stiffness. These proteins are all sensitive to reactive oxygen species, P_i_, and ADP accumulation, all of which accompany muscle fatigue and contribute to changes in active force [1].

Additionally, an increase in cytosolic calcium in skeletal muscle cells can also contribute to the changes in passive mechanical properties in late isotonic fatigue. Repeated contractions cause fragmentation of the RyR1 and calcium leakage from the sarcoplasmic reticulum in rodent and human skeletal muscle [64,65]. Even small increases in cytosolic calcium facilitate weak actin-myosin binding, a slight rise in the population of cross-bridges in the strong-binding state, and modulates titin stiffness, factors that can interfere with passive mechanical properties of muscle [66].

In summary, isotonic fatigue results in altered passive mechanical properties to muscle, which may be related to oxidative stress, accumulation of metabolites, such as P_i_ and ADP, and/or increased cytosolic calcium levels. However, if redox modifications are involved in the mechanism of altered viscoelastic properties during late isotonic fatigue, it is not through Nox2, since our results revealed no attenuation of energy loss and stiffness in skmNox2^KO^ mice.

### 4.4. Limitations

A primary limitation of this study is that experiments were conducted in muscle from male mice only. There are several indications that contractile function differs from male to female mice. However, adaptations of this protocol to an *ex vivo* preparation observed no differences in power or fatigue between healthy male and female mice [47]. Consideration of sex as a biological variable is critical to our understandings of fatigue in integrative physiology [67] and merits further study at the cellular, organ, and systemic/integrated level.

Furthermore, these investigations in murine diaphragm preparations can not immediately be translated to limb muscle preparations. The advantage of the diaphragm preparation stems from the thinness of the muscle that reduces the possibility of a hypoxic core during high activity. Adapting this protocol to *ex vivo* preparations in which the muscle remains fully perfused permits the application to limb muscles, as has recently been performed [47]. It is also important to iterate that mechanisms of fatigue may differ at different levels of intensity and with different protocols implemented. Indeed, one of the points of our discussion is to draw comparisons between this study and previous ones investigating the role of Nox2 with different approaches to study skeletal muscle fatigue. Our goal here were to develop a more physiologically relevant protocol to study mechanisms of fatigue, and highlight key areas of importance in experimental design.

## 5. Conclusions

Most practical uses of skeletal muscle involve muscle shortening and the development of mechanical power. Experiments studying mechanisms of muscle fatigue should implement an element of muscle shortening, as this contributes to the intensity of the protocol and may reveal mechanisms unaccounted for if just studying muscle force production through static contractions. Here, we describe an approach to measure muscle fatigue in isolated diaphragm strips using afterload contractions and provide comparisons of fatigue under different mechanical loads. Implementing this protocol, we found that knockout of skeletal muscle Nox2 prolongs fatigue development, with no apparent effects on recovery. Nox2 may be an important target for the attenuation of fatigue in athletes participating in multi-bout or -day sporting events, or in patients who suffer from accelerated fatigue as a consequence of their disease. However, chronically targeting Nox2 in healthy muscle impairs adaptations to exercise training [68].

## Supplementary Material

1

## Appendix A. Supplementary data

Supplementary data to this article can be found online at https://doi.org/10.1016/j.freeradbiomed.2025.08.045.
