# Supplementary material for: Skeletal muscle knockout of NAD(P)H oxidase 2 delays the development of isotonic diaphragm fatigue in mice
Source: Free Radic Biol Med. Author manuscript; Available in PMC 2026 Jun 14. (PMC13264669; doi:10.1016/j.freeradbiomed.2025.08.045)
Supplement: 1 [file NIHMS2181723-supplement-1.docx]

Figure S1 Comparison of different isotonic contractions. (A) Force- and position-time tracings of afterload contractions, in which the muscle develops the indicated load before shortening, against loads ranging from 15-70% F_max_. (B) Force- and position-time tracings of isotonic release contractions, in which the muscle is maximally activated isometrically before shortening against the indicated load.

**Figure S2. Comparison of tension-time index (TTI) between fatigue protocols.** A single diaphragm strip was subjected to four seconds of different fatigue protocols to make comparisons between the TTI of each protocol. (A) Isometric contractile protocol described in Loehr et al. [23] (B) Isometric contractile protocol described in Cheng et al. [53]. (C) Isotonic contractile protocol developed herein where the muscle shortens against 45% of the maximum force (F_max_).
